# In Vitro Activity of Essential Oils Against Planktonic and Biofilm Cells of Extended-Spectrum β-Lactamase (ESBL)/Carbapenamase-Producing Gram-Negative Bacteria Involved in Human Nosocomial Infections

**DOI:** 10.3390/antibiotics9050272

**Published:** 2020-05-25

**Authors:** Ramona Iseppi, Alessandro Di Cerbo, Piero Aloisi, Mattia Manelli, Veronica Pellesi, Cinzia Provenzano, Stefania Camellini, Patrizia Messi, Carla Sabia

**Affiliations:** 1 Department of Life Sciences, University of Modena and Reggio Emilia, Via G. Campi 287, 41125 Modena, Italy; ramona.iseppi@unimore.it (R.I.); 177187@studenti.unimore.it (V.P.); 46885@studenti.unimore.it (C.P.); stefania.camellini@studenti.unimore.it (S.C.); patrizia.messi@unimore.it (P.M.); 2 School of Biosciences and Veterinary Medicine, University of Camerino, Via Circonvallazione 93/95, 62024 Matelica, Italy; alessandro.dicerbo@unicam.it; 3 Laboratory of Clinical Chemistry and Microbiology, Hesperia Hospital, Via Arquà 80/A, 1125 Modena, Italy; paloisi@hesperia.it (P.A.); mmanelli@hesperia.it (M.M.)

**Keywords:** antibiotic resistance, ESBL, KPC, MBL, essential oils, biofilm

## Abstract

The aim of this study was to analyze the antibacterial activity of four essential oils (EOs), *Melaleuca alternifolia*, *Eucalyptus globulus*, *Mentha piperita,* and *Thymus vulgaris,* in preventing the development and spread of extended-spectrum β-lactamase (ESBL)-producing *Escherichia coli* and *Klebsiella pneumoniae*, metallo-beta-lactamase (MBL)-producing *Pseudomonas aeruginosa* and carbapenemase (KPC)-producing *Klebsiella pneumoniae*. A total of 60 strains were obtained from the stock collection from the Microbiology Laboratory of Hesperia Hospital, Modena, Italy. Twenty ESBL-producing *E. coli*, 5 *K. pneumoniae*, 13 KPC-producing *K. pneumoniae,* and 20 MBL-producing *P. aeruginosa* were cultured and reconfirmed as ESBL and carbapenamase producers. Polymerase chain reaction was used for the detection of genes responsible for antibiotic resistance (ESBL and KPC/MBL). Antibacterial activity of the EOs was determined using the agar disk diffusion assay, and minimal inhibitory concentrations (MICs) were also evaluated. Lastly, adhesion capability and biofilm formation on polystyrene and glass surfaces were studied in 24 randomly selected strains. *M. alternifolia* and *T. vulgaris* EOs showed the best antibacterial activity against all tested strains and, as revealed by agar disk diffusion assay, *M. alternifolia* was the most effective, even at low concentrations. This effect was also confirmed by MICs, with values ranging from 0.5 to 16 µg/mL and from 1 to 16 µg/mL, for *M. alternifolia* and *T. vulgaris* EOs, respectively. The EOs’ antibacterial activity compared to antibiotics confirmed *M. alternifolia* EO as the best antibacterial agent. *T. vulgaris* EO also showed a good antibacterial activity with MICs lower than both reference antibiotics. Lastly, a significant anti-biofilm activity was observed for the two EOs (*P < 0.05 and **P < 0.01 for *M. alternifolia* and *T. vulgaris* EOs, respectively). A good antibacterial and anti-biofilm activity of *M. alternifolia* and *T*. *vulgari*s EOs against all selected strains was observed, thus demonstrating a future possible use of these EOs to treat infections caused by ESBL/carbapenemase-producing strains, even in association with antibiotics.

## 1. Introduction

The improper and uncontrolled use of antibiotics in human and veterinary medicine resulted in the occurrence of multi drug resistant (MDR) strains, which have become a major health concern worldwide [1,2,3,4]. MDR microorganisms, both Gram-positive and Gram-negative bacteria, are those bacteria that acquired a non-susceptibility to one or more classes of antimicrobials as per in vitro tests [5,6]. The production of enzymes by extended-spectrum beta-lactamase (ESBL) and carbapenemase-producing Gram-negative bacteria is a problem in most of hospital facilities worldwide. The widespread use of antimicrobials, primarily antibiotics, and the transmissibility of resistance determinants mediated by plasmids, transposons, and gene cassettes in integrons contributed to the spread of resistance [7].

This problem of increasing resistance imposed the search for safe and effective factors that might be used to treat persistent bacterial infections. The severity and extent of diseases caused by these pathogens are also amplified when the pathogens are organized in biofilms, the most relevant structures responsible for persistent infections, that constitute a major challenge for microbiologists and clinicians. A biofilm is a microorganisms’ community that produces an extracellular matrix to attach itself to biotic or abiotic surfaces, embedded in an aqueous matrix of extracellular polymeric substances (EPS) [8]. Bacteria growing in biofilms are commonly even more resistant to antimicrobial agents and are protected from the host immune response, thus increasing chronic infections that are particularly difficult to nurse [9,10]. One mechanism of biofilm resistance to antimicrobial agents is the failure of an agent to penetrate the full depth of the biofilm. Biofilm resistance is supposed to be mediated by the ability of some of the biofilm cells that, undergoing nutrient restriction, can persist in a slow-growing or starved state. However, a recent theory proposed that at least some of the cells in a biofilm can adopt a distinct and protecting biofilm phenotype [11].

Experimental studies have already confirmed different pharmaceutical activities not only of chemical compounds, but also of many plant metabolites, such as polysaccharides, flavonoids, coumarins, glycosides, phenolic acids, saponins, and essential oils (EOs). Plant metabolites are a very interesting alternative for synthetic preparations, many of which are endowed with strong antimicrobial activity [12,13]. Antibacterial effects of EOs and their compounds (s-(+)-linalool, terpinen-4-ol carvacrol, eugenol) have been recently tested in many investigations against bacteria, both in planktonic and sessile forms [14,15,16].

EOs are mixtures of compounds obtained from spices, aromatic herbs, fruits, and flowers, and their antimicrobial properties against bacteria and fungi have long been known. Considering the increasing resistance phenomenon in pathogens, both in hospital and community settings, investigations on the antimicrobial activities, mode of action, and potential uses of EOs and their components have gained a new popularity for study [17]. EOs have already demonstrated antimicrobial activity [18,19,20,21], with additive and synergistic effects with antibiotics used against antibiotic resistant bacteria. Recent studies highlighted the synergistic role of EOs with other antimicrobials and how these associations could be used to improve the activity of some antibiotics, with consequent reduction of therapeutic doses [22,23,24].

The objective of the preliminary investigation was to firstly assess the antimicrobial activity of some EOs obtained/extracted from four different spices, *Melaleuca alternifolia* and *Eucalyptus globulus* (Myrtaceae Family) and *Mentha piperita* and *Thymus vulgaris* (Labiatae Family), against planktonic and biofilm cells of MDR Gram-negative bacteria such as *Escherichia coli (E. coli*), *Klebsiella pneumoniae* (*K. pneumoniae*), and *Pseudomonas aeruginosa (P. aeruginosa*), ESBL/carbapenemase-producing strains involved in human nosocomial infections and susceptible only to colistin, imipenem, meropenem, or ertapenem, depending on the bacterial strains.

## 2. Results

### 2.1. Phenotypic Identification of ESBLs and Carbapenamase

Gram-negative isolates were identified as *E. coli*, *K. pneumoniae,* and *P. aeruginosa* strains using Vitek-2 (bioMérieux, Florence, Italy). Out of the 60 strains analyzed, 27 (45%) strains showed an increase (5 mm) in the inhibition zone diameter for cefotaxime and ceftazidime in the presence of amoxicillin/clavulanic acid (AMC) compared to when these antibiotics were tested alone: These isolates were classified as ESBL producers. Moreover, thirty-three (55.6%) out of 60 strains tested for the NG-Test CARBA were positive, among these, thirteen (39.4%) were carbapenemase (KPC) and twenty (60.6%) metallo-beta-lactamase (MBL). 

### 2.2. Polymerase Chain Reaction and Sequencing of ESBL and Carbapenemase Genes

All the ESBL/carbapenamase-producing strains were directly sequenced by PCR and analyzed. In the ESBL isolates the following genes were found (species/number of isolates): blaCTX-M-15 (*E. coli*/15), blaTEM-52 (*E. coli*/ 5; *K. pneumoniae*/2), and blaCTX-M-1(*K. pneumoniae*/5), whereas in the carbapemase-producing strains, blaKPC-2 (*K. pneumoniae*/13), blaVIM-1 (*P. aeruginosa*/15), and blaVIM-2 (*P. aeruginosa*/5) genes were recovered.

### 2.3. Antibacterial Activity Evaluation of the Essential Oils

#### 2.3.1. Agar Disk Diffusion Assay

**Figure 1:**
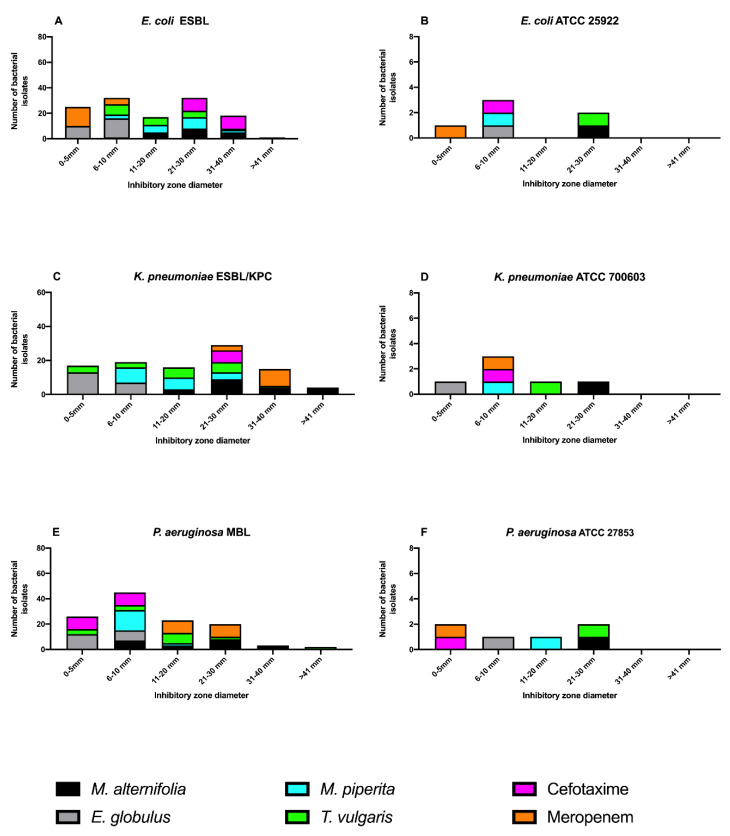
Antibacterial activity of essential oils (EOs) meropenem and cefotaxime by agar disc assay. Ranges of inhibitory zone diameter for *E. coli* ESBL (**A**), *E. coli* ATCC 25922 (**B**), *K. pneumoniae* ESBL/KPC (**C**), *K. pneumoniae* ATCC 700603 (**D**), *P. aeruginosa* MBL (**E**), and *P. aeruginosa* ATCC 27853 (**F**). ESBL: extended-spectrum β-lactamase; ATCC: American Type Culture Collection; KPC: carbapenemase; MBL: metallo-beta-lactamase.

The agar disk diffusion assay was employed as a screening test for the determination of antimicrobial activities of the EOs, by measuring the zone of inhibition of the bacteria growth (mm). The American Type Culture Collection (ATCC) strains were used as a positive control. The results revealed that *M. alternifolia* EO had a wide antibacterial spectrum and inhibited the growth of almost all tested strains (Figure 1). 

In particular, the inhibition zone ranged from 21 to 30 mm for *E. coli* (8 strains, 40%), *K. pneumoniae* (9 strains, 45.5%), and *P. aeruginosa* (8 strains, 40%) and from 31 to 40 mm for the remaining susceptible *E. coli* (5 strains, 25%), *K. pneumoniae* (4 strains, 20%), and *P. aeruginosa* (3 strains, 15%). A good antibacterial activity was also shown by *T. vulgaris* EO, with a range of inhibition zone from 21 to 30 mm for *E. coli* (9 strains, 45%), *K. pneumoniae* (4 strains, 20%) and *P. aeruginosa* (2 strains, 10%), whereas the remaining susceptible *E. coli* and *K. pneumoniae* showed a zone of inhibition ranging from 31 to 40 mm for 1 strain (5%) and 6 strains (30%), respectively. *M. piperita* EO exhibited an antibacterial activity similar to *T. vulgaris* EO, with an inhibition zone ranging from 6 to 20 mm. *E. globous* EO showed a very low activity against all bacterial strains, with inhibition zone values from 0 to 10 mm. The inhibition zone of the two antibiotics cefotaxime and meropenem against all clinical isolated strains confirmed their antibiotic-resistance. The American Type Culture Collection (ATCC) strains (*E. coli* ATCC 25922, *K*. *pneumoniae* ATCC 700603, and *P*. *aeruginosa* ATCC 27853) were sensitive to EOs such as the clinically isolated strains. No activity was detected for the negative control in any of the performed tests.

#### 2.3.2. Minimal Inhibitory Concentration (MIC)

**Figure 2:**
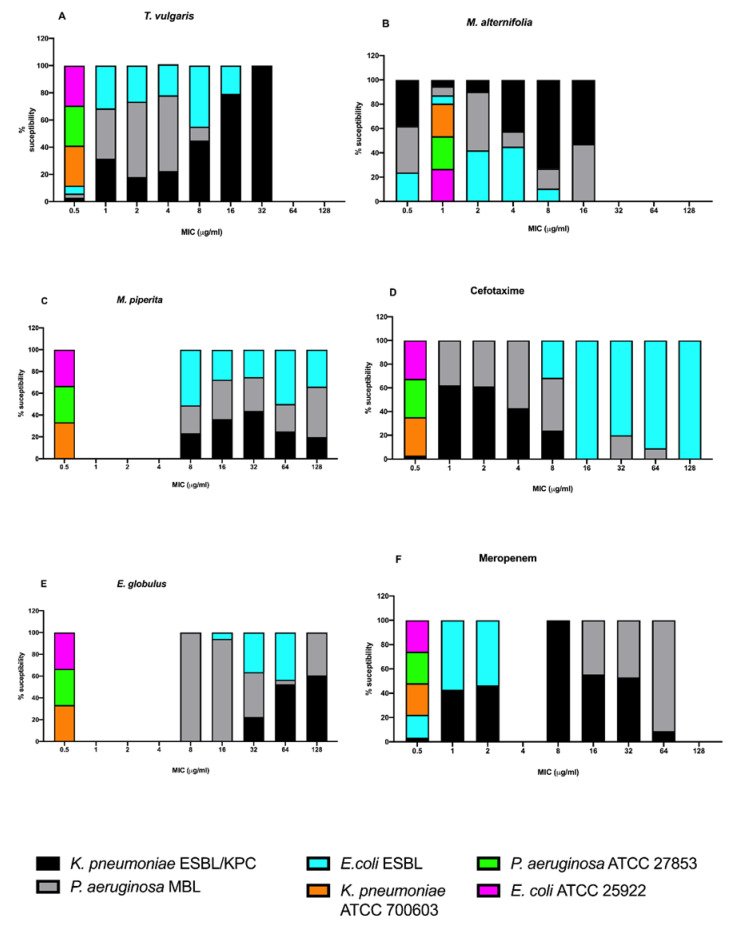
Minimal inhibitory concentration (MIC) for (**A**) *M. alternifolia*, (**B**) *M. piperita*, (**C**) *T. vulgaris,* (**D**) *E. globulus*, (**E**) cefotaxime, and (**F**) meropenem against *E. coli* ESBL, *E. coli* ATCC 25922, *K. pneumoniae* ESBL/KPC, *K. pneumoni*ae ATCC 700603, *P. aeruginosa* MBL, and *P. aeruginosa* ATCC 27853. All values were reported to a 100% total.

Figure 2 shows the minimal inhibitory concentration (MIC) values for EOs and for the two antibiotics (cefotaxime and meropenem) used. 

Before describing MIC values for each essential oil on each strain, the values were reported to a 100% total. A good antibacterial activity was observed for *M. alternifolia* and *T. vulgaris* EOs, as already observed with the agar disk diffusion assay. MIC values of *M. alternifolia* EO ranged from 0.5 to 16 µg/mL; in particular, a percentage of 55% of *K. pneumoniae*, 45% of *P. aeruginosa,* and 95% of *E. coli* presented MICs between 0.5 and 4 µg/mL. MIC values ranging from 1 to 16 µg/mL emerged for *T. vulgaris* EO, in a percentage of 90% for *K. pneumoniae* and *P. aeruginosa* and 85% for *E. coli.* Regarding *M. piperita* and *E. globulus*, the MIC ranges were from 8 to 128 µg/mL and from 32 to 64 µg/mL, respectively, with a percentage of 90% for *K. pneumoniae,* 80% of *P. aeruginosa,* and 95% of *E. coli*. Cefotaxime and meropenem MICs of all clinical isolates confirmed the antibiotic resistance patterns.

Lastly, the EOs’ antibacterial activity compared to antibiotics confirmed that *M. alternifolia* EO was the most active against all clinical strains even at low concentrations; *T. vulgaris* EO also showed a good activity with MICs lower than those of the two antibiotics used. Based on these results *M. alternifolia* and *T. vulgaris* EOs were selected for the antibiofilm study.

### 2.4. Biofilm Assay

Antibiofilm activities of *M. alternifolia* and *T. vulgaris* EOs against the 24 randomly selected strains are shown in Table 1, Table 2 and Table 3.

The 24 strains used in this part of the investigation were randomly selected as follows: 8 ESBL-producing *E. coli*; 2 ESBL-producing and 6 carbapenamase (KPC)-producing *K. pneumoniae*; and 8 carbapenamase- producing (MBL) *P. aeruginosa*. *E. coli* ATCC 25922, *K. pneumoniae* ATCC 700603, and *P. aeruginosa* ATCC 27853 were used as susceptible strains. As shown in Table 1, a significant decrease in biofilm production was observed for *E. coli* 7B, 7C, and ATCC 25922 treated with *M. alternifolia* EO and for 5A, 5M, 6I, 7D, 7E strains treated with *T. vulgaris* EO (*P < 0.05). A significant decrease in biofilm production was also observed (Table 2) for *K. pneumoniae* 1C–1G treated with *M. alternifolia* and for 1H, 1V, and ATCC 700603 strains treated with *T. vulgaris* EO, compared to positive control (*P < 0.05, **P < 0.01). Lastly, a significant decrease in biofilm production was observed (Table 3) for *P. aeruginosa* 3C and 3D treated with *M. alternifolia* and for strains 3E and ATCC 27858 strains treated with *T. vulgaris*, with respect to positive control (*P < 0.05). 

## 3. Discussion

Both the difficulties in the treatment of chronic infections and the increase of antibiotic resistant strains justify the increasing studies on new antibacterial compounds [25,26,27,28]. Interest is focused on naturally active compounds, capable of contrasting the MDR strains and reducing the adhesion and formation of their biofilms [29]. EOs’ inhibition of bacterial planktonic growth, including MDR strains, could be due to their ability to degrade membrane proteins and cell permeability [30]. Regarding biofilm formation, EOs are able to inhibit adhesion of bacterial cells at the first stage of biofilm formation, and some EOs also have the capability to inhibit the activity of quorum sensing inhibitors (QSI) [31], a bacterial intracellular communication system able to control the pathogenesis of many bacteria, including antibiotic resistance, expression of virulence factors, and biofilm formation. 

The aim of this research was to establish the antimicrobial activity of *M. alternifolia, E. globulus, M. piperita,* and *T. vulgaris* EOs against resistant bacteria and their biofilms. *M. alternifolia* and *T. vulgaris* EOs showed the best antibacterial activity against all strains tested, and a significant anti-biofilm capability also emerged for the two most active EOs used in the test, as reported by other authors [32,33,34,35]. Several essential oils have been studied to date for their antimicrobial properties, and the choice of the natural compounds is often based on what the environmental context can provide. In fact, within the same compound, there may be many variables that can influence its antimicrobial activity, for example, the soil where plants are grown and the extraction method. All information contributes to general and global knowledge, but study results have a more direct impact at the country level. There are many combinations of essential oils and of EOs and antibiotics to be investigated to find the best synergism to fight microorganisms responsible for infections (in particular when protected by biofilm) through the modulation of antibiotic resistance in the most problematic strains. More information will help to reduce the gap between the lack of new synthetic molecules and the problem of antibiotic resistance, which has considerable clinical and economic impact. Many researchers are trying to acquire and share their results on this vast and important topic, as recently highlighted in a WHO document (2019) that lists antibiotic resistance among the ten biggest plagues worldwide.

*M. piperita* EO displayed an antibacterial activity similar to that of *T. vulgaris* for the inhibition in the bacterial growth zone (agar well diffusion assay), but lower MIC values. *E. globulus* EO presented a very low antibacterial activity, so that EO and the *M. piperita* EO were not considered for further evaluations.

The data of MIC values confirmed the good activity of *M. alternifolia* and *T. vulgaris* EOs against all resistant strains with respect to antibiotics, and the MICs of the two EOs were lower than those of the antibiotics. The anti-mature biofilm evaluation also showed the capability of *M. alternifolia* and *T. vulgaris* EOs to counteract MDR strains in sessile forms. The antibacterial activity of *M. alternifolia* EO is attributed mainly to terpinen-4-ol, which is the major component of the oil and exhibits a favorable hydrophobic/hydrophilic profile [33]. *T. vulgaris* EO is characterized by phenolic compounds, such as thymol and carvacrol, and hydrocarbons, such as p-cymene and γ-terpinene [14,15,16]. In particular, phenolic compounds are well-known for their strong antibacterial activity, since they are capable of disintegrating bacterial outer membranes.

The major antibacterial mechanism of *M. alternifolia* and *T. vulgaris* EOs is the morphological alterations of permeability and the integrity of bacterial cell walls and membranes that lead to leakage of intracellular materials, such as electrolytes, ATP, proteins, and DNA materials [36]. 

Interestingly, EOs have also been used as cleaning liquid for disinfecting medical equipment and surfaces and are effective in controlling nosocomial infections [37]. In addition, they are used as aerosols in operating blocks and waiting rooms for air cleaning to limit contamination [38]. Their pleasant smell provides a pleasant feeling of psychic comfort for patients [39]. In a study from Benameur et al., the susceptibility of bla_ESBL_-producing *Enterobacteriaceae* to Slovakian *T. vulgaris* EOs with or without the antibiotic cefotaxime was assessed [40]. The authors reported the synergistic interaction of the EOs in combination with the antibiotic against bla*_S_*_HV-12_-producing MDR *E. coli* and an additive effect against ESBL-producing MDR *Enterobacter cloacae*. 

## 4. Materials and Methods 

### 4.1. Bacterial Strains and Phenotypic Identification 

A total of 60 strains of *K. pneumoniae, P. aeruginosa,* and *E. coli* were obtained from the bacterial glycerol stocks of Microbiology Laboratory of Hesperia Hospital, Modena, Italy. Sites of isolation included urine, rectal swabs, and respiratory tracts. The microorganisms included 25 *E. coli*, 5 ESBL-producing *K. pneumoniae*, 13 KPC-producing *K. pneumoniae,* and 20 MBL-producing *P. aeruginosa*. All strains from the stock were subcultured, and the identification of species and antimicrobial susceptibility testing was reconfirmed using the Vitek 2 system and AST-GN041 card (bioMerieux, Florence, Italy). The ESBL-producing isolates were further reconfirmed by the phenotypic double-disc synergy test (DDST), using both cefotaxime and ceftazidime alone and in combination with clavulanic, according to the Clinical and Laboratory Standards Institute [41]. We applied the NG-Test CARBA 5 immunochromatographic assay (NG Biotech, Guipry, France) for detecting the KPC, OXA-48-like, VIM, IMP, and NDM isolates. 

### 4.2. Polymerase Chain Reaction and Sequencing of ESBL and Carbapenemase Genes

Bacterial isolates confirmed for their capacity to produce ESBLs were further analyzed by PCR. DNA was extracted using a standard heat lysis protocol [42]. An overnight culture broth (1.5 mL) was centrifuged at 14,000 rpm for 5 min, and we re-suspended the bacterial pellet in 500 mL of distilled water. The cells were lysed by boiling them for 10 min at 95 °C, centrifuged at 14,000 rpm for 10 min, and finally 2 μL of the supernatant were used as a template for PCR. ESBL genes (bla_TEM_, bla_SHV_, and bla_CTX_-_M_) and carbapenamase genes (bla_KPC_; bla_IMP_, bla_VIM_, bla_OXA-48-like_, and bla_NDM_) were detected, as previously reported [43,44]. PCR-positive amplicons were purified with the PCR Purification Kit according to the manufacturer’s instructions (Qiagen, Milan, Italy) and directly sequenced using amplification primers on the 3130 Genetic Analyzer (Applied Biosystem, Milan, Italy). Purification and sequencing were carried out by Genex s.r.o. (CZ, Czech Republic). Sequence alignment and analysis were performed online using the BLAST program of the National Center for Biotechnology Information (www.ncbi.nlm.nih.gov).

### 4.3. Antibacterial Activity Evaluation of the Essential Oils

#### 4.3.1. Agar Disk Diffusion Assay

Essential oils (EOs) of *M. alternifolia, E. globulus, M. piperita,* and *T. vulgaris* were purchased from Merck Life Science SRL, Milan, Italy. These oils were selected based on a literature survey and their use in traditional medicine. Quality of the oils was ascertained to be more than 98% pure. The preliminary determination of the antibacterial activity of the four EOs against all isolated bacteria was carried out by using the agar disk diffusion assay, according to the standard procedure of the Clinical and Laboratory Standards Institute [41]. Plates containing Muller Hinton Agar (MHE, bioMérieux, Florence, Italy) were uniformly inoculated with 100 µL of 10^6^ CFU/mL of each strain suspension. Then, sterile disks of 6 mm in diameter, containing 10 µL of each EO, were placed on the agar surfaces. After incubation at 37 °C for 24 h, a clear zone of inhibition of the bacterial growth, expressed in millimeters (mm), was measured to quantify the EOs antibacterial activity [45]. Cefotaxime (5 μg) and meropenem (10 µg) discs were used as the positive control for *E. coli*, *K. pneumoniae,* and *P. aeruginosa*. A sterilized physiological saline solution (5 μL) was used as negative control. The classified Gram-negative bacteria (ATCC-American Type Culture Collection) *E. coli* ATCC 25922, *K. pneumoniae* ATCC 700603, and *P. aeruginosa* ATCC 27853 were used as positive control.

#### 4.3.2. Minimum Inhibitory Concentration (MIC) Determination 

The MIC evaluation was determined against all the microbial strains following the guidelines of the Clinical and Laboratory Standards Institute [41], with slight modifications. The assay was performed in sterile 96-well microplates by dispensing into each well 95 µL of tryptic soy broth, (TSB, bioMerieux, Florence, Italy) and 5 µL of bacterial suspensions, to final inoculums concentrations of 10^6^ CFU/mL. Each EO was diluted in TSB containing 0.5% Tween 80 (*v*/*v*) and 100 μL was added to wells to give final EO concentrations ranging from 0.25 to 512 μL/mL [46]. The last well, containing 195 μL of nutrient broth and 5 μL of bacterial strain without EO was used as negative control. The antibiotics cefotaxime and meropenem diluted in nutrient broth with strains added were used as positive control. The plates were incubated at 37 °C for 24 h. The MIC was defined as the lowest concentration of the EO that inhibited visible growth of the tested microorganisms after optical density (OD) measured at 570 nm, using a microtiter plate reader. The MIC values were expressed as μg/mL, by considering the density value for each EO. All the experiments were performed in triplicate.

### 4.4. Essential Oils’ Activity on Biofilm

The antibiofilm activity of two EOs, *M. alternifolia*, and *T. vulgaris*, chosen on the basis of the best antibacterial activity seen in the previous assays, was tested on 2-day-old pre-formed biofilms of 24 strains, randomly selected as follows: 8 ESBL-producing *E. coli*; 2 ESBL-producing and 6 carbapenamase (KPC)-producing *K. pneumoniae;* and 8 carbapenamase-producing (MBL) *P. aeruginosa*. As susceptible strains, *E. coli* ATCC 25922, *K. pneumoniae* ATCC 700603, and *P. aeruginosa* ATCC 27853 were used. The mature biofilm was obtained by growing the strains on 96-well polystyrene microtiter plates inoculated with 200 µL of 18-h-old bacterial culture containing a cell count of approximately 10^6^ CFU/mL. The TSB medium was refreshed every 24 h, and after biofilm formation, the medium was gently aspirated, and plates were washed three times with a sterile phosphate-buffered saline solution (PBS, pH 7.2) to remove planktonic bacteria. The two EOs and the two antibiotics cefotaxime and meropenem, used as positive control, were added at the respective MIC concentration and incubated for 24 h at 37 °C. The residual biofilm was determined by the crystal violet staining method [47]. For fixation of the biofilms, 150 μL of methanol for 15 min was added, and the supernatant was removed again. Then, 150 μL of crystal violet (CV) solution at 0.1% was added to each well and, 15 min later, the excess dye was removed by washing the plates three times with sterile PBS. The bound crystal violet was released by adding 200 µL of 33% acetic acid followed by incubation for 10 min at room temperature. The optical density (OD) was measured at 570 nm using a microplate reader (Sunrise Tecan, Grödig, Austria). TSB with bacterial culture added was used as the positive control and all determinations were performed in triplicate.

### 4.5. Statistical Analysis

All the experiments were carried out in triplicate, and the bacterial count was performed on three plates. All data are presented as the means ± SD. A Kruskal–Wallis test with Dunn’s multiple comparisons test was used to analyze differences among positive results obtained using *M. alternifolia* and *T. vulgaris* EOs, meropenem, and cefotaxime treatment for each strain of each species. All statistical analyses were performed with GraphPad Prism 8 (GraphPad Software Inc., San Diego, CA, USA). *P < 0.05 was considered significant.

## 5. Conclusions

In conclusion, the results that emerged from the present investigation and other existing data on essential oils show the wide range of antimicrobial activity against MDR bacteria. These results may be considered as a potential starting-point for additional studies on the activity for application of these natural compounds in drugs, food, and cosmetics, in combination with nanomaterials and antibiotics, as newer and more efficient antimicrobials able to closely interact with microorganisms.

## Figures and Tables

**Table 1:** *E. coli* biofilm values expressed as optical density with mean ± standard deviation (* *P* < 0.05).

Strain	Positive	*M. alternifolia*	*T. vulgaris*	Meropenem	Cefotaxime
*E. coli* 5A	2.19 ± 0.07	0.61 ± 0.11	0.56 ± 0.03 *	0.87 ± 0.03	1.92 ± 0.06
*E. coli* 5M	2.17 ± 0.01	0.76 ± 0.05	0.64 ± 0.08 *	0.93 ± 0.03	1.88 ± 0.07
*E. coli* 5Z	2.04 ± 0.03	0.43 ± 0.10	0.43 ± 0.10	0.83 ± 0.05	1.88 ± 0.04
*E. coli* 6I	1.89 ± 0.09	0.68 ± 0.01	0.44 ± 0.09 *	0.87 ± 0.07	1.86 ± 0.07
*E. coli* 7B	1.91 ± 0.06	0.25 ± 0.06 *	0.47 ± 0.11	0.85 ± 0.08	1.84 ± 0.08
*E. coli* 7C	1.92 ± 0.04	0.34 ± 0.06 *	0.45 ± 0.12	0.84 ± 0.08	1.83 ± 0.11
*E. coli* 7D	1.95 ± 0.06	0.45 ± 0.07	0.23 ± 0.09 *	0.92 ± 0.08	1.89 ± 0.11
*E. coli* 7E	1.95 ± 0.06	0.45 ± 0.07	0.23 ± 0.09 *	0.92 ± 0.08	1.89 ± 0.11
*E. coli* ATCC 25922	2.11 ± 0.09	0.58 ± 0.08 *	0.33 ± 0.08	0.55 ± 0.13	0.39 ± 0.12

**Table 2:** *K. pneumoniae* biofilm values expressed as optical density with mean ± standard deviation (* *P* < 0.05, ** *P* < 0.01).

Strain	Positive	*M. alternifolia*	*T. vulgaris*	Meropenem	Cefotaxime
*K. pneumoniae* 1B	1.91 ± 0,06	0.71 ± 0.05	0.27 ± 0.03	1.42 ± 0.04	1.49 ± 0.05
*K. pneumoniae* 1C	1.88 ± 0.10	0.61 ± 0.04 *	0.86 ± 0.04	1.45 ± 0.05	1.39 ± 0.02
*K. pneumoniae* 1D	1.95 ± 0.15	0.35 ± 0.11 *	0.31 ± 0.17 *	1.16 ± 0.21	0.92 ± 0.05
*K. pneumoniae* 1E	1.99 ± 0.10	0.22 ± 0.09 *	0.44 ± 0.08	0.95 ± 0.13	1.00 ± 0.14
*K. pneumoniae* 1F	2.01 ± 0.10	0.37 ± 0.08 *	0.54 ± 0.08	0.96 ± 0.14	0.86 ± 0.10
*K. pneumoniae* 1G	2.01 ± 0.11	0.55 ± 0.13 *	0.78 ± 0.03	1.79 ± 0.10	1.78 ± 0.05
*K. pneumoniae* 1H	2.05 ± 0.10	0.43 ± 0.07	0.21 ± 0.04 *	1.80 ± 0.07	1.68 ± 0.04
*K-pneumoniae* 1V	2.91 ± 0.02	0.73 ± 0.09	0.45 ± 0.06 **	0.90 ± 0.08	1.04 ± 0.11
*K. pneumoniae* ATCC 700603	2.04 ± 0.07	0.19 ± 0.01	0.12 ± 0.003 *	0.17 ± 0.06	0.34 ± 0.12

**Table 3:** *P. aeruginosa* biofilm values expressed as optical density with mean ± standard deviation (* *P* < 0.05).

Strain	Positive	*M. alternifolia*	*T. vulgaris*	Meropenem	Cefotaxime
*P. aeruginosa* 3A	1.85 ± 0.46	0.24 ± 0.12	0.35 ± 0.08	1.84 ± 0.07	1.75 ± 0.09
*P. aeruginosa* 3B	2.03 ± 0.07	0.26 ± 0.07	0.46 ± 0.09	2.03 ± 0.06	1.92 ± 0.07
*P. aeruginosa* 3C	2.15 ± 0.14	0.36 ± 0.08 *	0.45 ± 0.07	2.14 ± 0.12	2.43 ± 0.15
*P. aeruginosa* 3D	2.85 ± 0.13	0.22 ± 0.04 *	0.98 ± 0.07	2.73 ± 0.01	2.84 ± 0.76
*P. aeruginosa* 3E	2.86 ± 0.10	0.56 ± 0.10	0.35 ± 0.08 *	2.71 ± 0.09	2.84 ± 0.08
*P. aeruginosa* 3F	2.59 ± 0.22	0.55 ± 0.10	0.26 ± 0.08 *	2.71 ± 0.09	2.85 ± 0.08
*P. aeruginosa* 3M	2.18 ± 0.42	0.50 ± 0.06	0.25 ± 0.07 *	2.44 ± 0.07	1.86 ± 0.09
*P. aeruginosa* 3P	1.85 ± 0.81	0.54 ± 0.08	0.28 ± 0.04 *	2.45 ± 0.08	1.74 ± 0.04
*P. aeruginosa* ATCC 27858	2.28 ± 0.09	0.55 ± 0.06	0.26 ± 0.06 *	0.67 ± 0.10	0.75 ± 0.04

## References

[1] Zaman S.B., Hussain M.A., Nye R., Mehta V., Mamun K.T., Hossain N. (2017). A Review on Antibiotic Resistance: Alarm Bells are Ringing. Cureus.

[2] Iseppi R., Di Cerbo A., Messi P., Sabia C. (2020). Antibiotic Resistance and Virulence Traits in Vancomycin-Resistant Enterococci (VRE) and Extended-Spectrum beta-Lactamase/AmpC-producing (ESBL/AmpC) Enterobacteriaceae from Humans and Pets. Antibiotics (Basel).

[3] Di Cerbo A., Canello S., Guidetti G., Laurino C., Palmieri B. (2014). Unusual antibiotic presence in gym trained subjects with food intolerance; a case report. Nutr. Hosp..

[4] Palmieri B., Di Cerbo A., Laurino C. (2014). Antibiotic treatments in zootechnology and effects induced on the food chain of domestic species and, comparatively, the human specie. Nutr. Hosp..

[5] Falagas M.E., Koletsi P.K., Bliziotis I.A. (2006). The diversity of definitions of multidrug-resistant (MDR) and pandrug-resistant (PDR) Acinetobacter baumannii and Pseudomonas aeruginosa. J. Med. Microbiol..

[6] Magiorakos A.P., Srinivasan A., Carey R.B., Carmeli Y., Falagas M.E., Giske C.G., Harbarth S., Hindler J.F., Kahlmeter G., Olsson-Liljequist B. (2012). Multidrug-resistant, extensively drug-resistant and pandrug-resistant bacteria: An international expert proposal for interim standard definitions for acquired resistance. Clin. Microbiol. Infect.

[7] Putman M., van Veen H.W., Konings W.N. (2000). Molecular properties of bacterial multidrug transporters. Microbiol. Mol. Biol. Rev..

[8] Tapia-Rodriguez M.R., Bernal-Mercado A.T., Gutierrez-Pacheco M.M., Vazquez-Armenta F.J., Hernandez-Mendoza A., Gonzalez-Aguilar G.A., Martinez-Tellez M.A., Nazzaro F., Ayala-Zavala J.F. (2019). Virulence of Pseudomonas aeruginosa exposed to carvacrol: Alterations of the Quorum sensing at enzymatic and gene levels. J. Cell Commun. Signal.

[9] Maita P., Boonbumrung K. (2014). Association between biofilm formation of Pseudomonas aeruginosa clinical isolates versus antibiotic resistance and genes involved with biofilm. J. Chem. Pharm. Res..

[10] Macia M.D., Rojo-Molinero E., Oliver A. (2014). Antimicrobial susceptibility testing in biofilm-growing bacteria. Clin. Microbiol. Infect.

[11] Singh S., Singh S.K., Chowdhury I., Singh R. (2017). Understanding the Mechanism of Bacterial Biofilms Resistance to Antimicrobial Agents. Open Microbiol. J..

[12] Burt S. (2004). Essential oils: Their antibacterial properties and potential applications in foods—A review. Int. J. Food Microbiol..

[13] Silva N., Fernandes Júnior A. (2010). Biological properties of medicinal plants: A review of their antimicrobial activity. J. Venom. Anim. Toxins Incl. Tropical Dis..

[14] Pei R.S., Zhou F., Ji B.P., Xu J. (2009). Evaluation of combined antibacterial effects of eugenol, cinnamaldehyde, thymol, and carvacrol against E. coli with an improved method. J. Food Sci..

[15] Aelenei P., Miron A., Trifan A., Bujor A., Gille E., Aprotosoaie A.C. (2016). Essential Oils and Their Components as Modulators of Antibiotic Activity against Gram-Negative Bacteria. Medicines (Basel).

[16] Mohamed S.H., Mohamed M.S.M., Khalil M.S., Azmy M., Mabrouk M.I. (2018). Combination of essential oil and ciprofloxacin to inhibit/eradicate biofilms in multidrug-resistant Klebsiella pneumoniae. J. Appl. Microbiol..

[17] Nouzha H., Souhila B., Manel M., Yasmine O., Lamraoui A. (2018). Screening for antibacterial activity of some essential oils and evaluation of their synergistic effec. Int. J. Biosci..

[18] Sakkas H., Gousia P., Economou V., Sakkas V., Petsios S., Papadopoulou C. (2016). In vitro antimicrobial activity of five essential oils on multidrug resistant Gram-negative clinical isolates. J. Intercult. Ethnopharmacol..

[19] Valdivieso-Ugarte M., Gomez-Llorente C., Plaza-Diaz J., Gil A. (2019). Antimicrobial, Antioxidant, and Immunomodulatory Properties of Essential Oils: A Systematic Review. Nutrients.

[20] Iseppi R., Brighenti V., Licata M., Lambertini A., Sabia C., Messi P., Pellati F., Benvenuti S. (2019). Chemical Characterization and Evaluation of the Antibacterial Activity of Essential Oils from Fibre-Type Cannabis sativa L. (Hemp). Molecules.

[21] Condo C., Anacarso I., Sabia C., Iseppi R., Anfelli I., Forti L., de Niederhausern S., Bondi M., Messi P. (2020). Antimicrobial activity of spices essential oils and its effectiveness on mature biofilms of human pathogens. Nat. Prod. Res..

[22] Yap P.S., Lim S.H., Hu C.P., Yiap B.C. (2013). Combination of essential oils and antibiotics reduce antibiotic resistance in plasmid-conferred multidrug resistant bacteria. Phytomedicine.

[23] Cheesman M.J., Ilanko A., Blonk B., Cock I.E. (2017). Developing New Antimicrobial Therapies: Are Synergistic Combinations of Plant Extracts/Compounds with Conventional Antibiotics the Solution?. Pharmacogn. Rev..

[24] Safaei-Ghomi J., Ahd A.A. (2010). Antimicrobial and antifungal properties of the essential oil and methanol extracts of Eucalyptus largiflorens and Eucalyptus intertexta. Pharmacogn. Mag..

[25] Ait Said L., Zahlane K., Ghalbane I., El Messoussi S., Romane A., Cavaleiro C., Salgueiro L. (2015). Chemical composition and antibacterial activity of Lavandula coronopifolia essential oil against antibiotic-resistant bacteria. Nat. Prod. Res..

[26] Predoi D., Iconaru S.L., Buton N., Badea M.L., Marutescu L. (2018). Antimicrobial Activity of New Materials Based on Lavender and Basil Essential Oils and Hydroxyapatite. Nanomaterials (Basel).

[27] Borges A., Lopez-Romero J.C., Oliveira D., Giaouris E., Simões M. (2017). Prevention, removal and inactivation of Escherichia coli and Staphylococcus aureus biofilms using selected monoterpenes of essential oils. J. Appl. Microbiol..

[28] El-Shouny W.A., Ali S.S., Sun J., Samy S.M., Ali A. (2018). Drug resistance profile and molecular characterization of extended spectrum beta-lactamase (ESbetaL)-producing Pseudomonas aeruginosa isolated from burn wound infections. Essential oils and their potential for utilization. Microb. Pathog.

[29] Vazquez-Armenta F.J., Hernandez-Onate M.A., Martinez-Tellez M.A., Lopez-Zavala A.A., Gonzalez-Aguilar G.A., Gutierrez-Pacheco M.M., Ayala-Zavala J.F. (2020). Quercetin repressed the stress response factor (sigB) and virulence genes (prfA, actA, inlA, and inlC), lower the adhesion, and biofilm development of L. monocytogenes. Food Microbiol..

[30] Ortega-Ramirez L.A., Gutiérrez-Pacheco M.M., Vargas-Arispuro I., González-Aguilar G.A., Martínez-Téllez M.A., Ayala-Zavala J.F. (2020). Inhibition of Glucosyltransferase Activity and Glucan Production as an Antibiofilm Mechanism of Lemongrass Essential Oil against Escherichia coli O157:H7. Antibiotics.

[31] Quave C.L., Horswill A.R. (2014). Flipping the switch: Tools for detecting small molecule inhibitors of staphylococcal virulence. Front Microbiol..

[32] Carson C.F., Hammer K.A., Riley T.V. (2006). Melaleuca alternifolia (Tea Tree) oil: A review of antimicrobial and other medicinal properties. Clin. Microbiol. Rev..

[33] Oliva A., Costantini S., De Angelis M., Garzoli S., Bozovic M., Mascellino M.T., Vullo V., Ragno R. (2018). High Potency of Melaleuca alternifolia Essential Oil against Multi-Drug Resistant Gram-Negative Bacteria and Methicillin-Resistant Staphylococcus aureus. Molecules.

[34] Ferrini A.M., Mannoni V., Aureli P., Salvatore G., Piccirilli E., Ceddia T., Pontieri E., Sessa R., Oliva B. (2006). Melaleuca alternifolia essential oil possesses potent anti-staphylococcal activity extended to strains resistant to antibiotics. Int. J. Immunopathol. Pharmacol..

[35] Sienkiewicz M., Kowalczyk E., Wasiela M. (2012). Recent Patents Regarding Essential Oils and the Significance of their Con-stituents in Human Health and Treatment. Recent Patents Anti-Infective Drug Discov..

[36] Chouhan S., Sharma K., Guleria S. (2017). Antimicrobial Activity of Some Essential Oils-Present Status and Future Perspectives. Medicines (Basel).

[37] Warnke P.H., Lott A.J., Sherry E., Wiltfang J., Podschun R. (2013). The ongoing battle against multi-resistant strains: In-vitro inhibition of hospital-acquired MRSA, VRE, Pseudomonas, ESBL E. coli and Klebsiella species in the presence of plant-derived antiseptic oils. J. Craniomaxillofac Surg..

[38] De Billerbeck V.G. (2007). Huiles essentielles et bactéries résistantes aux antibiotiques. Phytothérapie.

[39] El Asbahani A., Miladi K., Badri W., Sala M., Ait Addi E.H., Casabianca H., El Mousadik A., Hartmann D., Jilale A., Renaud F.N. (2015). Essential oils: From extraction to encapsulation. Int. J. Pharm..

[40] Benameur Q., Gervasi T., Pellizzeri V., Pľuchtová M., Tali-Maama H., Assaous F., Guettou B., Rahal K., Gruľová D., Dugo G. (2019). Antibacterial activity of Thymus vulgaris essential oil alone and in combination with cefotaxime against blaESBL producing multidrug resistant Enterobacteriaceae isolates. Nat. Prod. Res..

[41] CLSI (2019). Performance Standards for Antimicrobial Susceptibility Testing.

[42] Perez-Perez F.J., Hanson N.D. (2002). Detection of plasmid-mediated AmpC beta-lactamase genes in clinical isolates by using multiplex PCR. J. Clin. Microbiol..

[43] Kim J., Jeon S., Lee B., Park M., Lee H., Lee J., Kim S. (2009). Rapid Detection of Extended Spectrum β-Lactamase (ESBL) for Enterobacteriaceae by use of a Multiplex PCR-based Method. Infect Chemother..

[44] Nordmann P., Poirel L., Carrer A., Toleman M.A., Walsh T.R. (2011). How to detect NDM-1 producers. J. Clin. Microbiol..

[45] Klancnik A., Piskernik S., Jersek B., Mozina S.S. (2010). Evaluation of diffusion and dilution methods to determine the antibacterial activity of plant extracts. J. Microbiol. Methods.

[46] Şahin F., Güllüce M., Daferera D., Sökmen A., Sökmen M., Polissiou M., Agar G., Özer H. (2004). Biological activities of the essential oils and methanol extract of Origanum vulgare ssp. vulgare in the Eastern Anatolia region of Turkey. Food Control.

[47] Stepanovic S., Vukovic D., Hola V., Di Bonaventura G., Djukic S., Cirkovic I., Ruzicka F. (2007). Quantification of biofilm in microtiter plates: Overview of testing conditions and practical recommendations for assessment of biofilm production by staphylococci. APMIS.

